# On the role of hypocrisy in escaping the tragedy of the commons

**DOI:** 10.1038/s41598-021-97001-3

**Published:** 2021-09-02

**Authors:** Amos Korman, Robin Vacus

**Affiliations:** 1 The French-Israeli Laboratory on Foundations of Computer Science, IRL FILOFOCS, CNRS, UP7, TAU, HUJI, WIS International Research Laboratory, Tel-Aviv, Israel; 2 The Research Institute on the Foundations of Computer Science (IRIF), CNRS and University of Paris, Paris, France

**Keywords:** Evolution, Climate sciences, Ecology, Environmental social sciences, Engineering, Mathematics and computing

## Abstract

We study the emergence of cooperation in large spatial public goods games. Without employing severe social-pressure against “defectors”, or alternatively, significantly rewarding “cooperators”, theoretical models typically predict a system collapse in a way that is reminiscent of the “tragedy-of-the-commons” metaphor. Drawing on a dynamic network model, this paper demonstrates how cooperation can emerge when the social-pressure is mild. This is achieved with the aid of an additional behavior called “hypocrisy”, which appears to be cooperative from the external observer’s perspective but in fact hardly contributes to the social-welfare. Our model assumes that social-pressure is induced over both defectors and hypocritical players, though the extent of which may differ. Our main result indicates that the emergence of cooperation highly depends on the extent of social-pressure applied against hypocritical players. Setting it to be at some intermediate range below the one employed against defectors allows a system composed almost exclusively of defectors to transform into a fully cooperative one quickly. Conversely, when the social-pressure against hypocritical players is either too low or too high, the system remains locked in a degenerate configuration.

## Introduction

The “tragedy-of-the-commons” metaphor, popularized by Hardin in 1968^1^, aims to capture situations in public goods systems where self-interested individuals behave contrary to the common good by depleting or spoiling the shared resource. In the 21st century, this metaphor finds relevance in several of our global environmental challenges^2,3^, where the shared resource can be considered, depending on the context, as an aspect of the ecosystem. For example, excessive beef consumption by a substantial number of individuals induces vast livestock production that degrades air and water quality and causes a considerable increase in greenhouse gas emissions^4^. Conversely, our environment would significantly benefit if a large portion of individuals in the population would self-restraint the amount of beef they consume. Therefore, improving our understanding of the emergence of cooperation in public goods systems goes beyond the purely theoretical interest and may prove to be of practical importance.

Theoretical studies on the emergence of cooperation typically assume that players act according to few stereotyped behaviors, the most common being “defector”, and “cooperator”^5–9^. A cooperator pays an energetic cost to produce a benefit *b* for others, whereas a defector does not contribute anything but also does not pay any energetic cost. In recent years, significant attention has been devoted to study the impact of the populations’ structure on the emergence of cooperation^5,10–12^. These works assume that players are organized over a fixed network, with the vertices representing the players and the edges representing reciprocal relations between neighbors. Naturally, the dynamics of the system strongly depend on the mutual relations between neighboring players.

For example, several of the works on cooperation in structured populations assume that the benefit *b* produced by a cooperative player is shared equally by its neighbors. For such a model, Ohtsuki et al. showed that cooperation emerges when the ratio between the benefit per neighbor and the cost of producing it exceeds a certain threshold^5^. However, large public goods games, especially those on the scale that affects the environment, exhibit a very different framework of reciprocity^13–17^. Rather than being shared by immediate neighbors, the benefit *b* is shared by all players, practically making the marginal per-capita return gain (MPCR) negligible compared to the cost of cooperating. This violates the condition for the evolution of cooperation based on reciprocity^5–9^ suggesting that cooperation in large public goods games might be difficult to achieve without considering other factors, such as rewards or punishments.

It is well-known that global cooperation can emerge when players severely punish their neighboring defectors (or, alternatively, significantly reward their cooperating neighbors)^6,14,18–22^. However, inducing severe punishments on others may be costly, and hence reaching high levels of social-pressure is by itself a non-trivial problem, often referred to in the literature as the *second-order free riders* problem^23–28^. A crucial parameter in the second-order problem is the cost of punishing, which may be correlated to the extent of punishment^29^. Clearly, when the cost exceeds a certain threshold, people would avoid punishing non-cooperators. However, when the cost is low, other factors, such as reputation considerations, can subsume the cost, ultimately making punishing beneficial^30–32^. It is therefore of interest to study the emergence of cooperation in the presence of moderate punishments or mild social-pressure.

Specifically, we are interested in a regime of social-pressure that is high enough to maintain an already cooperative system, but is insufficient to transform a system that initially includes a large number of defectors into a cooperative one. To illustrate this, let us consider the context of recycling and an imaginary person named Joe. When almost all of Joe’s neighbors are recycling (i.e., cooperating), the social-pressure cost they induce on him can accumulate to overshadow the burden cost of recycling and incentivize him to also recycle. Conversely, when almost all of Joe’s neighbors are not recycling (i.e., defecting), the burden of recycling may exceed the overall social-pressure, effectively driving Joe to defect. This raises a natural question:

*How can a system that utilizes mild social-pressure escape the tragedy-of-the-commons when it is initially composed mostly of defectors?*

The aforementioned recycling abstraction includes two extreme behaviors: defection and cooperation. Another type of generic behavior is *hypocrisy*^25,26,33–36^, which was also experimentally studied in^37,38^. In our interpretation, a hypocritical player pretends to be cooperative in order to reduce the social-pressure that it might experience as a defector, and, at the same time, avoids the high energetic cost incurred by a cooperator. To pretend to be a cooperator, a hypocritical player must invest a small amount of energy in contributing to the social welfare, as well as mimic the behavior of cooperators towards their peers. This means that such players, similarly to cooperators, also induce mild social-pressure. In other words, and in contrast to *disguising* players as in^39^, hypocritical players actively “demand” cooperation from others, as part of their strategy to hide their low investment in the social-welfare.

It was previously suggested that hypocritical behavior can incentivize global cooperation^26,27^. However, in these works, similarly to many other papers on the emergence or evolution of cooperation based on reciprocity^5–9^, the dynamics heavily relies on the assumption that players gain substantially from the presence of nearby cooperators. As mentioned, this assumption is hardly justifiable in large-scale public goods scenarios such as the ones we consider.

## Results

We consider public goods games played iteratively over a fixed connected network. The vertices of the network represent the players and the edges represent neighboring connections^5,10–12^. The dynamics evolve in discrete rounds. In each round, each player chooses a behavior that minimizes its cost, where the player’s cost is affected by its own behavior and the behaviors of its neighbors.

Our main model includes three behavior types, namely, defection, hypocrisy, and cooperation, in which those who hardly contribute to the social welfare, i.e., defector and hypocritical players, face the risk of being caught and punished by their neighbors who are non-defectors. The level of risk together with the extent of punishment is captured by a notion that we call *“social-pressure”*. The main result is that adjusting the level of social-pressure employed against hypocritical players compared to the one employed against defectors can have a dramatic impact on the dynamics of the system. Specifically, letting the former level of social-pressure be within a certain range below the latter level, allows the system to quickly transform from being composed almost exclusively of defectors to being fully cooperative. Conversely, setting the level to be either too low or too high locks the system in a degenerate configuration.

As mentioned, our main model assumes that non-defectors induce mild social-pressure on the defectors among their neighbors. This implicitly assumes that inducing the corresponding social-pressure is beneficial (e.g., allows for a social-upgrade), although other explanations have also been proposed^21^. To remove this implicit assumption we also consider a generalized model, called the *two-order model*, which includes costly punishments. Consistent with previous work on the second-order problem, e.g.,^23,25–27,36,40^, this model distinguishes between first-order cooperation, that corresponds to actions that directly contribute to the social welfare, and second-order cooperation, that corresponds to applying (costly) social-pressure, or punishments, on others. As in the main model, the level of punishment employed against first-order defectors may differ from that employed against second-order defectors. We identify a simple criteria for the emergence of cooperation: For networks with minimal degree $\Delta$, cooperation emerges when two conditions hold. The first condition states that the cost $\alpha _2$ of employing punishments against second-order defectors should be smaller than the corresponding punishment $\beta _2$ itself, i.e., $\alpha _2<\beta _2$. The second condition states that the cost $\alpha _ 1$ of employing punishments against first-order defectors should be smaller than the corresponding punishment $\beta _1$ times the minimal number of neighbors, i.e., $\alpha _ 1<\beta _1\cdot \Delta$. The second condition is also a necessary condition for the emergence of cooperation in the two-order model.

### The main model

The model considers two extreme behaviors, namely, *cooperation* (*c*) and *defection* (*d*), and an additional intermediate behavior, called *hypocrisy* (*h*). The system starts in a configuration in which almost all players, e.g., $99\%$, are defectors (see “Methods”). Execution proceeds in discrete rounds. The cost of a player depends on its own behavior and on the behavior of its neighbors. All costs are evaluated at the beginning of each round, and then, before the next round starts, each player chooses a behavior that minimizes its cost (breaking ties randomly), given the current behavior of its neighbors. In other words, we assume that players greedily choose their best behavior, given the current configuration. In our simulations, we also consider a relaxed version, where players choose the best behavior with high probability, and with small probability choose an arbitrary behavior. In contrast to many previous works on cooperation in networks^5–9^, we assume that benefits from altruistic acts are negligible (i.e., the MPCR is zero), so that a player does not gain anything when others cooperate.

The cost of a player *u* with a behavior type $i\in \{d,h,c\}$ is composed of two components: the *energetic cost* $E_i$ associated with the contribution to the social welfare, and the *social-pressure cost* $S_i(u)$ it faces, that is:

$$\begin{aligned} {\mathcal{{C}}}_i(u)=E_i+S_i(u). \end{aligned}$$

We assume that the energetic cost of a defector is 0, and the energetic cost of a cooperator is 1, where the value of 1 is chosen for normalization:

$$\begin{aligned} E_{d}=0\quad {\text{ and }}\quad E_{c}=1. \end{aligned}$$

A hypocritical player produces the minimal social welfare required to pretend to be cooperative. Hence, we assume that

$$\begin{aligned} 0<E_h<1, \end{aligned}$$

thinking of $E_h$ as closer to 0 than to 1.

As mentioned above, we focus on relatively mild social-pressure induced by cooperative players, aiming to improve their social status. Since hypocritical players aim to appear similar to cooperators from the perspective of an external observer, we assume that they too induce social-pressure on their neighbors. Defectors, on the other hand, do not induce any social-pressure since such an enhancement of the social status is not justified for them. In principle, cooperators and hypocritical players might induce different levels of social-pressure, yet, for the sake of simplicity, we assume that they induce the same extent of social-pressure. This assumption is further justified by the fact that a player *u* cannot distinguish its hypocritical neighbors from its cooperative neighbors, hence, *u*’s calculation of the social-pressure is evaluated assuming all of its non-defector neighbors are cooperators.

Formally, we assume that the possible social-upgrade gain associated with cooperators or hypocritical players as a result of applying social-pressure is already taken into account when calculating the energetic costs $E_c$ and $E_h$. Since we assume that this gain is small, it hardly perturbs the cost, keeping the energy consumption as the dominant component.

Implicitly, we think of the social-pressure cost incurred by a player *u* as the product of two factors: (1) the risk of being caught, which is assumed to be proportional to the number of *u*’s neighbors inducing social-pressure, and (2) a fixed penalty paid when caught, which depends on *u*’s behavior. The product of the risk and penalty represents the expected punishment in the next round, if behaviors remain the same.

Cooperators are assumed to pay zero penalty, and are hence effectively immune to social-pressure:

$$\begin{aligned} S_{c}(u)=0. \end{aligned}$$

Conversely, the social-pressure induced over defectors and hypocritical players is non-zero. For a given round, let $\Delta _{\bar{d}}(u)$ denote the number of neighbors of *u* which are non-defectors at that round. The social-pressure cost induced over a defector, and respectively, a hypocritical, player *u* is:

$$\begin{aligned} S_{d}(u)=\rho _{d}\cdot \Delta _{\bar{d}}(u),\quad {\text{ respectively, }} \quad S_{h}(u)=\rho _{h}\cdot \Delta _{\bar{d}}(u), \end{aligned}$$

where $\rho _{d} >0$, respectively $\rho _{h} >0$, represents the social-pressure induced over a defector, respectively a hypocritical, from one neighboring non-defector. Note that when comparing the social-pressure incurred by defectors versus hypocritical players, both the risk of being caught and the extent of punishment are expected to be different. Indeed, since hypocritical players pretend to be cooperators, their risk of being caught is expected to be lower than that of defectors. Moreover, after being caught, the respected punishment of a defector might be different than that of a hypocritical player, depending on the social norms. Altogether, here we focus on the regime where $\rho _h < \rho _d$, since otherwise, becoming a defector is always more beneficial than becoming a hypocritical.

To sum up, at a given round, the total cost incurred by a player *u* is:

$$\begin{aligned} {\mathcal{{C}}}(u) = {\left\{ \begin{array}{ll} 1 &{}\quad {\text{ if }}\,\, u\,\, {\text{ is a cooperator, }} \\ \rho _{d} \cdot \Delta _{\bar{d}}(u) &{}\quad {\text{ if }}\,\, u\,\, {\text{ is a defector, }} \\ E_h + \rho _{h} \cdot \Delta _{\bar{d}}(u) &{}\quad {\text{ if }}\,\, u\,\, {\text{ is hypocritical. }} \end{array}\right. } \end{aligned}$$

Before stating our main result, we recall few standard definitions in graph-theory^41^. The *diameter* of a network *G*, denoted ${\text {diam}}(G)$, is the maximal distance between any pair of players (see “Methods”). A network is $\Delta$-*regular*, if every player has precisely $\Delta$ neighbors. Theorem 1 below assumes that the underlying network is $\Delta$-regular. However, this theorem can be generalized to arbitrary networks with minimal degree $\Delta$ (see SI, Theorem 6).

#### Theorem 1

*Consider a*$\Delta$*-regular networkGwithnplayers. Assume that*

1 $$\begin{aligned} ({1-E_h})/{\Delta }<\rho _{h}<\rho _{d}-E_h. \end{aligned}$$

*Then, with probability at least*$1-\frac{1}{c^n}$, *for some constant*$c>1$, *in at most*$3 \cdot {\text {diam}}(G)+1$*rounds, the system will be in a configuration in which all players are cooperative, and will remain in this configuration forever*.

**Figure 1:**
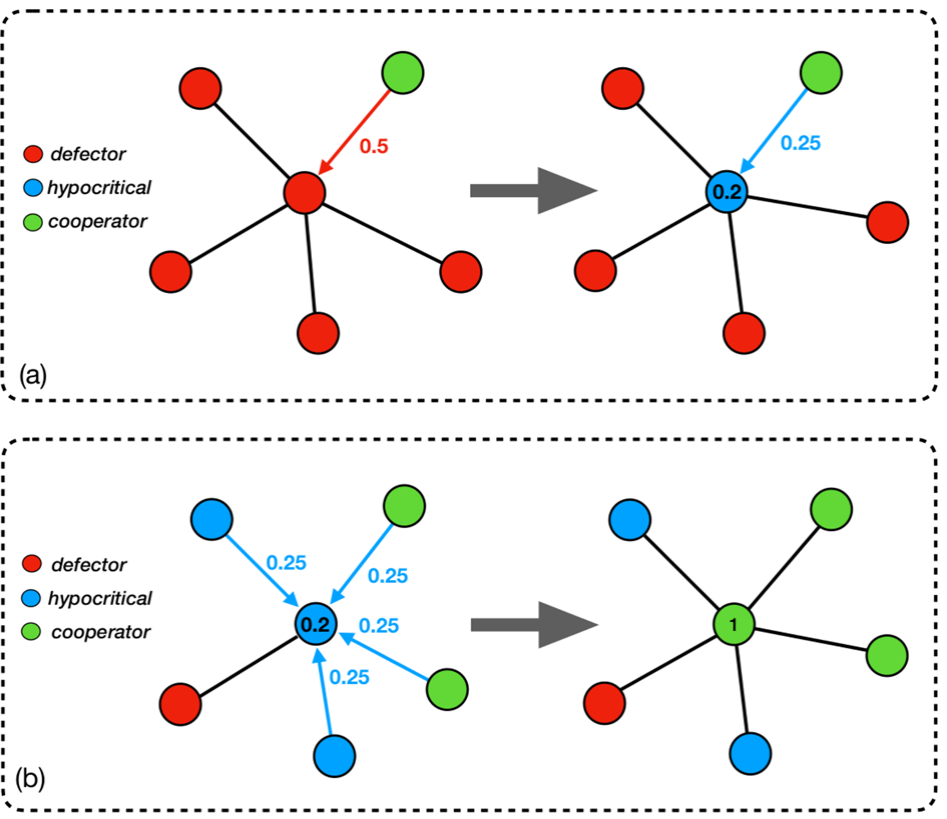
The two stages of the dynamics. The direction of the red and blue arrows indicates the direction of the social-pressure applied on the player occupying the central vertex. Cooperative players pay an energetic cost of $E_c=1$ and are immune to social-pressure. A defector player pays a social-pressure cost of $\rho _{d}=0.5$ per non-defector neighbor. A hypocritical player pays social-pressure cost of $\rho _{h}=0.25$ per non-defector neighbor, and an energetic cost of $E_h=0.2$. (**a**) First stage: defectors become hypocritical players. A defector player (central vertex on the left) has one non-defector neighbor (in this case, a cooperator), implying that its social-pressure cost is $\rho _{d}=0.5$. Therefore, that player would prefer to be hypocritical (right), paying only $0.25+0.2=0.45$. (**b**) Second stage: hypocritical players become cooperators. Here, a hypocritical player (central vertex on the left) is surrounded by four non-defector neighbors. In this case, the social-pressure accumulates to favor cooperation (right).

The formal proof of Theorem 1 appears in the SI, Section B. Intuitively, the main idea behind it is as follows. When the extent of social-pressure against hypocritical players is moderate, that is, when $\rho _h$ satisfies Eq. (1), the transition process can be divided into two stages. At the first stage, since the punishments of hypocritical players are sufficiently lower than those of defectors, specifically, $\rho _{h}<\rho _{d}-E_h$, or equivalently $\rho _{h}+E_h<\rho _{d}$, the presence of at least one neighboring non-defector *u* makes a hypocritical player pay less than a defector. In this case, *u*’s neighbors would become non-defectors at the next round (Fig. 1a). Although this does not necessarily imply that *u* itself remains a non-defector in the next round, it is nevertheless possible to show that the proportion of hypocritical players gradually increases on the expense of defectors. Note that at this point, the social welfare may still remain low, since hypocritical players hardly contribute to it. However, the abundance of non-defectors increases the overall social-pressure in the system. In particular, since the social-pressure on hypocritical players is also not too mild, specifically $(1-E_h)/{\Delta }<\rho _{h}$, or equivalently $1<\rho _{h}\Delta +E_h$, the presence of many neighboring non-defectors can magnify it up to the point that the total cost incurred by a hypocritical player surpasses the energetic cost of being a cooperator (Fig. 1b). At this second stage, cooperators prevail over both defectors and hypocritical players, and so the system converges to a cooperative configuration.

Conversely, severely punishing hypocritical players diminishes the prevalence of such players, preventing the system from escaping the initial degenerate configuration. Contrariwise, incurring too mild social-pressure towards hypocritical players would prevent the second stage of the dynamics. In particular, if $\rho _h < (1-E_h)/\Delta$, or equivalently, if $E_h + \rho _h \Delta < 1$, then a player would always prefer to be hypocritical over being cooperative (even when all its neighbors induce social-pressure). In this case, the system would remain degenerative since the population would consist of a combination of defectors and hypocritical players.

**Figure 2:**
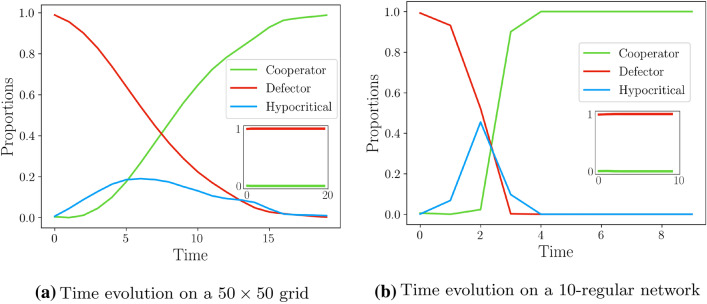
Evolution of cooperation in grids and random 10-regular networks. **(a)** Corresponds to a $50\times 50$ grid network, and (**b**) corresponds to a random 10-regular network with 1000 vertices. Both simulations start with a configuration in which 99% of players are defectors. (**a**) and (**b**) show how the population evolves over time (number of rounds). The chosen parameters satisfy the assumption in Eq. (1). The insets show the population’s evolution when hypocritical behavior is not available to the agents. See “Methods” for more details.

To illustrate the dynamics we conducted simulations over several types of networks. Figure 2 shows how the population evolves over time, when considering a grid network (Fig. 2a) and a random 10-regular network (Fig. 2b). The chosen parameters satisfy the assumption in Eq. (1). In both dynamics, the role of hypocritical behavior as a transitory state, essential to achieving cooperation, is well illustrated by the initial peak of hypocritical players, preceding the rise of cooperative players. Moreover, if hypocritical behavior is disabled (see “Methods”), then the system is unable to escape the defective state (insets).

**Figure 3:**
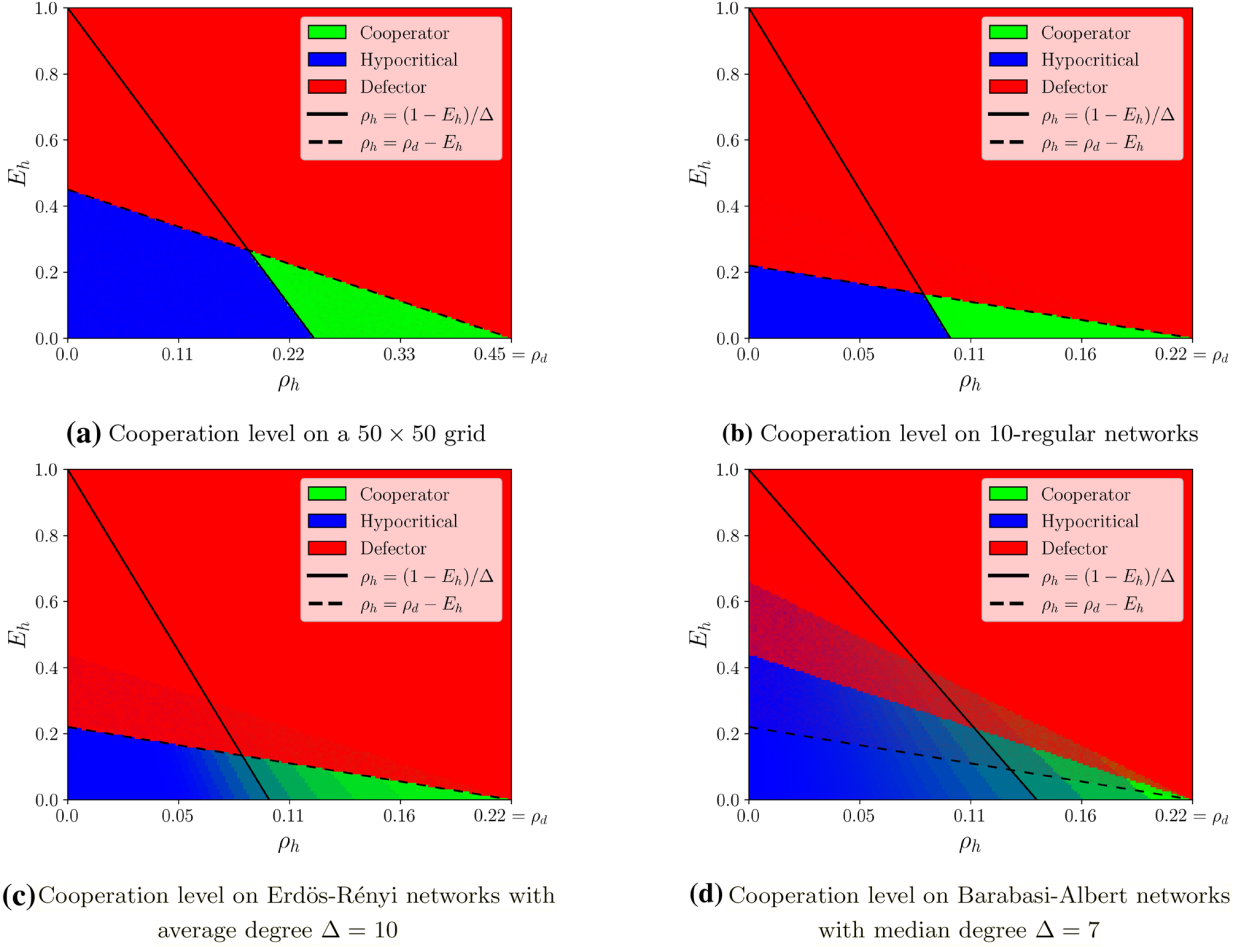
Emergence of cooperation on various networks. The figure depicts the steady-state levels of cooperation on different network families. (**a**) Corresponds to a $50\times 50$ grid network, (**b**) corresponds to random 10-regular networks with 1000 vertices, (**c**) corresponds to Erdös–Rényi networks with 1000 vertices and parameter $p=1/100$, and (**d**) corresponds to Barabási–Albert networks with 1000 vertices and parameter $m=5$. All simulations start with a configuration in which $99\%$ of players are defectors. In all figures, for each couple $(\rho _h,E_h)$, a pixel is being drawn, whose red (resp. green, blue) component corresponds to the average proportion of defectors (resp. cooperators, hypocrites) at steady state. See “Methods” for more details.

Figure 3 depicts the steady-state configuration, when hypocritical players experience different levels of energetic cost ($E_h$) and social-pressure ($\rho _h$). This is illustrated on a grid network (Fig. 3a), random 10-regular networks (Fig. 3b), Erdös–Rényi networks with average degree 10 (Fig. 3c), and a Barabasi Albert networks with median degree 7 (Fig. 3d). The figures indicate that for small values of $\rho _h$ and $E_h$, hypocritical behavior is, unsurprisingly, dominant: punishments deter defectors, but are insufficient to incentivize cooperation. For moderate values of $E_h$, this phenomenon changes when $\rho _h$ enters the range specified in Theorem 1. Then, when $\rho _h$ increases further, the system remain defective. The correspondence to Theorem 1 is striking in Fig. 3a–c, whereas it is slightly more moderate in Fig. 3d. Recall that Theorem 1 considers $\Delta$-regular networks, and therefore directly applies to grid networks and random regular networks, as simulated in Fig. 3a,b, respectively. Moreover, although a typical Erdös–Rényi network is not regular, the degrees of its vertices are relatively concentrated around the average degree, justifying the similarity between the results in Fig. 3b,c. For Barabasi Albert networks (Fig. 3d) the average degree is not a good representative for the typical degree since these networks are power-law. Hence, we drew the line corresponding to $\rho _h=(1-E_h)/\Delta$, taking $\Delta$ to be the median degree, which was in this case roughly 7. Even though many vertices in the network have a smaller degree than the median degree, high levels of cooperation emerge in the region specified by Theorem 1.

**Figure 4:**
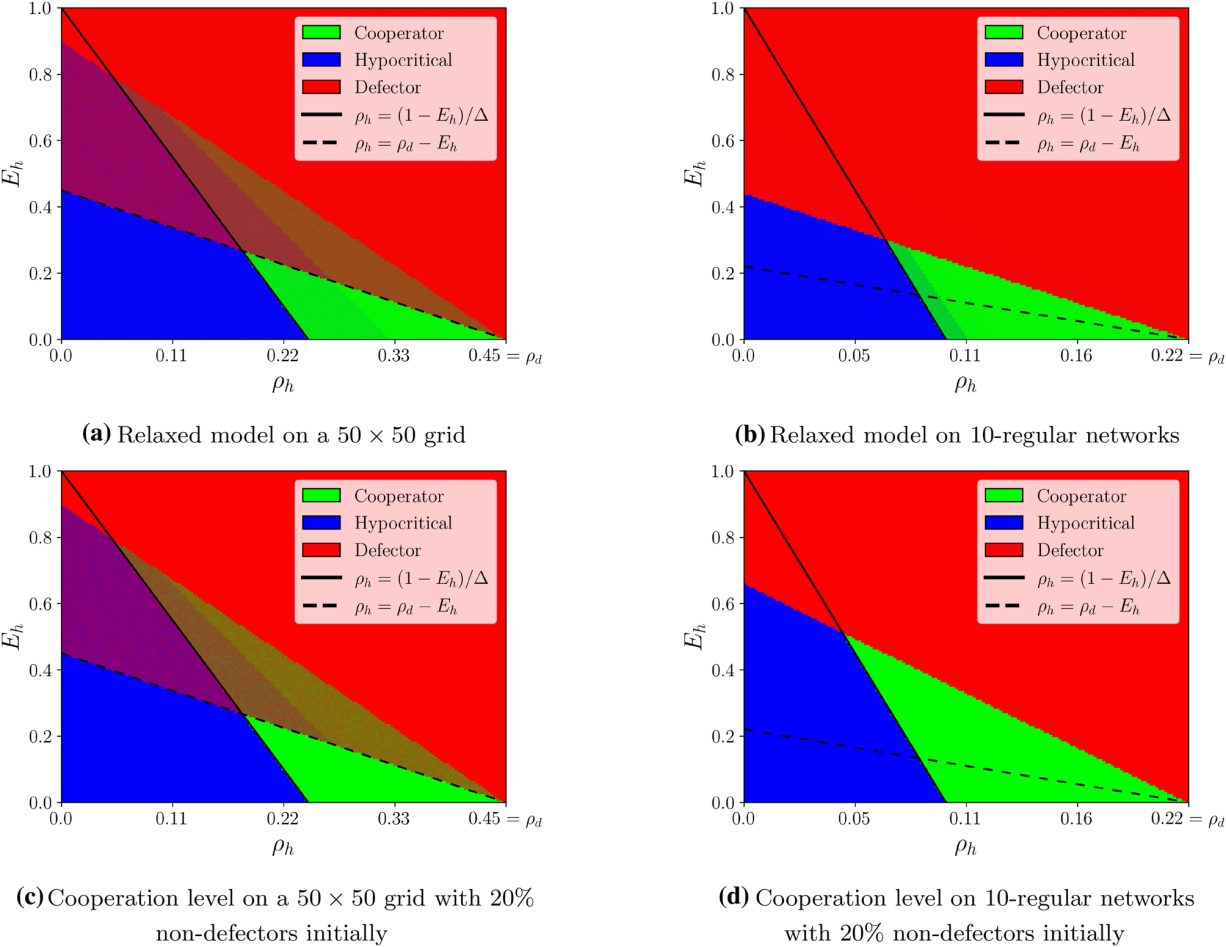
Relaxed model on grids and random 10-regular networks. The figure depicts simulation results using relaxed versions of the main model. In (**a**) and (**b**) the greediness assumption in the decision making process is relaxed, allowing for some “irrationality” (see “Methods” for more details). In (**c**) and (**d**), the initial configuration contains $80\%$ defectors, instead of $99\%$ as used in Fig. 3. The plots in (**a**) and (**c**) correspond to a $50\times 50$ grid network and should be compared with Fig. 3a, whereas (**b**) and (**d**) correspond to random 10-regular networks with 1000 vertices and should be compared with Fig. 3b.

Consistent with Theorem 1, Fig. 3 considers the case that players behave in a fully greedy fashion while having perfect knowledge regarding their costs. To check if this assumption is impactful, we also simulated a more noisy variant of our model, in which each player chooses the behavior that minimizes its cost with probability 0.95, and otherwise chooses a behavior uniformly at random. This relaxed model yields more mixed populations at steady-state, as indicated in Fig. 4a regarding a grid network and in Fig. 4b regarding random 10-regular networks. As another relaxation, we also simulated the case that the initial configuration is not overwhelmingly composed of defectors. Specifically, in Fig. 4c (grid network) and 4d (random 10-regular networks) we assumed that initially $80\%$ of the players are defectors, instead of $99\%$ as used in Fig. 3. Not surprisingly, this relaxation enhances cooperation. Indeed, comparing Fig. 4c to Fig. 3a, and comparing Fig. 4d to Fig. 3b, we observe that for each of these networks, the corresponding regime of cooperation includes the one that emerges when there are more defectors initially. Overall, in all the relax versions in Fig. 4 we see that the necessity of the condition $\rho _h > (1-E_h)/\Delta$ to the emergence of cooperation is still respected. However, the other condition mentioned in Theorem 1, namely, $\rho _h < \rho _d - E_h$ appears to be more sensitive to randomness. Indeed, and especially for the cases of random $\Delta$-regular networks, cooperation emerges also for larger values of $\rho _h$.

### A generalized model with costly punishments

We next describe a different, more general model, termed the *two-order model*, that includes costly punishments. We then show how the second-order problem is solved in this model for a certain regime of parameters.

As the name suggests, the two-order model includes two levels of cooperation. Players engaged in *first-order cooperation* incur an energetic cost to produce a benefit for other individuals, whereas players engaged in *second-order cooperation* induce costly punishments on other individuals whenever they fail to cooperate (on any order). The two orders of cooperation are not mutually exclusive, that is, a player can cooperate (or not cooperate) on one of the two orders or on both.

Similarly to the main model, players are organized over a connected network *G*. A *behavior* for Player *u* is defined as a couple of indicator functions $(\chi _1(u),\chi _2(u))$, with the convention that $\chi _1(u) = 1$ if *u* cooperates on the first-order (and 0 if it defects), and $\chi _2(u) = 1$ if *u* cooperates on the second-order (and 0 if it defects).

The cost incurred by a player is divided into two components. We denote by $\alpha _1 > 0$ the cost associated with first-order cooperation (this is analogues to the energetic cost in the main model), while $\alpha _2 > 0$ refers to the cost of second-order cooperation, that is, the cost of incurring punishments. A player *u* such that $\chi _2(u) = 1$ induces a *social-pressure cost* on each of its neighbors, whenever these fail to cooperate, at any order. As in the main model, the extent of this social-pressure may differ depending on whether it is applied against first-order defectors or second-order defectors. Specifically, we denote by $\beta _1$ the social-pressure cost paid by a first-order defector, and by $\beta _2$ the social-pressure cost paid by a second-order defector (fully defecting players pay both). Formally, denoting by $\Delta _2(u)$ the number of neighbors of *u* which are cooperating on the second-order, that is, $\Delta _2(u) = |\{v\, { is\, a\, neighbor\, of}\, u, \chi _2(v) = 1 \}|$, the total cost paid by *u* equals:

2 $$\begin{aligned} {\mathcal{{C}}}(u) = \chi _1(u) \alpha _1 + \chi _2(u) \alpha _2 + (1-\chi _1(u)) \Delta _2(u) \beta _1+ (1-\chi _2(u)) \Delta _2(u) \beta _2. \end{aligned}$$

Let us name each of the four behaviors, and recap their cost:

- *a cooperator* ($$\chi _1(u) = 1 , \chi _2(u) = 1$$) pays $$\alpha _1 + \alpha _2$$,
- *a defector* ($$\chi _1(u) = 0 , \chi _2(u) = 0$$) pays $$\Delta _2(u) (\beta _1+ \beta _2)$$,
- *a hypocritical* ($$\chi _1(u) = 0 , \chi _2(u) = 1$$) pays $$\alpha _2 + \Delta _2(u) \beta _1$$,
- *a private cooperator* ($\chi _1(u) = 1 , \chi _2(u) = 0$) pays $\alpha _1 + \Delta _2(u) \beta _2$.

As in the main model, the system starts in a configuration in which almost all players, e.g., $99\%$, are defectors (see “Methods”). The execution proceeds in discrete synchronous rounds. The costs of each player are evaluated at the beginning of each round, and then, before the next round starts, each player chooses a behavior that minimizes its cost (breaking ties randomly), given the current behavior of its neighbors.

The theorem below assumes that the underlying network is $\Delta$-regular. However, as in the case of Theorem 1, the theorem can be generalized to arbitrary networks with minimal degree $\Delta$ (SI, Theorem 13).

#### Theorem 2

*Consider a*$\Delta$-*regular networkGwithnplayers undergoing the two-order model. Assume that the following two conditions hold*.

- *Condition (i)*$$\alpha _2 < \beta _2$$, and
- *Condition (ii)*$$\alpha _1 < \Delta \beta _1$$.

*Then, with probability at least*$1-\frac{1}{c^n}$, *for some constant*$c>1$, *in at most*$3 \cdot {\text {diam}}(G)+1$*rounds, the system will be in a configuration in which all players are cooperative, and will remain in this configuration forever*.

The formal proof of Theorem 2 appears in the SI, Section C. Intuitively, the proof starts by showing that for the regime of parameters satisfying Conditions (*i*) and (*ii*), after the first round, no player ever chooses to be a private cooperator. The proof proceeds by showing that for this regime of parameters, the dynamics of the two-order model can be translated to the dynamics of the main model for the regime of parameters satisfying Eq. (1). In other words, the proof of Theorem 2 is based on a reduction to Theorem 1.

## Discussion

This paper proposes a simple idealized network model that demonstrates how cooperation can emerge, even when the MPCR is zero, and even when the extent of social-pressure is low. Our results highlight the possible social role that might be played by hypocritical behavior in escaping the tragedy-of-the-commons. The main finding is that setting the level of social-pressure towards this behavior to be at a specific intermediate range allows to quickly transform an almost completely defective system into a fully cooperative one. Our model, like any model, neglects many of the real-life complexity parameters. Nevertheless, the insight we discovered sheds new light on the possibility of emergent cooperation. In particular, our results suggest that those who wish to influence others in the context of environmental preservation should rethink their relation to their hypocritical acquaintants.

## Methods

For two players *u* and *v* in *G*, let $d_G(u,v)$ denote the *distance* between *u* and *v*, that is, the number edges on the shortest path linking *u* to *v* in *G*. The maximal distance between any pair of players, i.e., the *diameter*, is denoted by ${\text {diam}}(G) = \max _{u,v \in G} d_G(u,v)$.

The initial configuration is governed by a given fixed $0<\epsilon <1$, which is independent from the underlying network. In the main model, each player is initially set to be a defector with probability $1-\epsilon$, a hypocritical with probability $\epsilon /2$, and a cooperative with probability $\epsilon /2$. Similarly, in the two-order model, each player is initially chosen to be a defector, with probability $1-\epsilon$, and, otherwise, with probability $\epsilon$ it chooses one of the three remaining behaviors with equal probability, i.e., $\epsilon /3$. To demonstrate the strength of the emergence of cooperation, we consider $\epsilon$ as very small; for example, in each of our simulations (except the ones corresponding to Fig. 4c,d), we took $\epsilon =0.01$, which means that initially, $99\%$ of the population were defectors, $0.5\%$ were hypocritical, and $0.5\%$ were cooperators.

We simulated the dynamics of the main model using the C++ language. Figures were obtained using the Python library “Matplotlib”. In Figs. 3a, 4a,c and 2a we used a $50 \times 50$, 4-regular, torus grid. In Figs. 3b, 4b,d and 2b, we used random 10-regular networks with 1000 vertices. To sample such a network, we gradually increased the number of edges, by pairing the vertices of degree less than 10 uniformly at random, until it became not possible anymore; then we discarded the few “left-overs” if necessary. As a consequence, the sampled networks have sometimes slightly less than 1000 vertices, but are always 10-regular by construction.

For Fig. 3c, we constructed Erdös–Rényi networks with 1000 vertices, taking each edge with probability $p=0.01$. For Fig. 3d, we constructed Barabási–Albert networks with 1000 vertices using the parameter $m=5$. To sample such a network, we started with an *m*-clique, and then added each new vertex by attaching it to *m* existing vertices chosen at random, with a probability proportional to their current degree.

When running the time-simulations on the grid in Fig. 2a, we took $E_h = 0.1$, $\rho _d = 0.45$, and $\rho _h = 0.23$. In Fig. 2b, the time-simulation was executed on a single random 10-regular network, using the parameters $E_h = 0.1$, $\rho _d = 0.22$, and $\rho _h = 0.11$. For both cases these parameters satisfy the constraints in Eq. (1). The insets show the evolution of the population when hypocritical behavior is disabled. This means that each player must choose between cooperation and defection only, and that in the initial configuration, each player is a defector with probability $1-\epsilon$, and a cooperator with probability $\epsilon$. The setting remains otherwise unchanged.

In both Figs. 3 and 4, the results of the simulations are presented for 150 values of $E_h$ and 150 values of $\rho _h$, with $E_h \in [ 0,1 ]$ and $\rho _h \in [0,\rho _d]$. For each couple $(E_h,\rho _h)$, a pixel is drawn at the appropriate location, whose RGB color code corresponds to the proportions of defectors (red), cooperators (green), and hypocritical players (blue) in steady-state—that is, after T rounds. These proportions have been averaged over N repetitions, with each time a new starting configuration, and, a new network. For the grid, we set $T=20, N=10$, whereas for the other networks, we took $T=10, N=100$.

Figure 4a,b were obtained similarly to Fig. 3a,b, respectively, except that players did not choose greedily their behaviors for the next round. Instead, at each round, each player chose a behavior that minimizes its cost (breaking ties randomly) with probability 0.95, and otherwise chose a behavior uniformly at random. Figure 4c,d were obtained similarly to Fig. 3a,b, respectively, except that the initial proportion of non-defectors was $20\%$ (instead of $1\%$), i.e., we took $\epsilon = 0.2$ (instead of 0.01).

All the experiments mentioned in this paper were numerical simulations. Specifically, they do not involve any real participant.

## Supplementary Information

Supplementary Information.
