# Supplementary material for: On the role of hypocrisy in escaping the tragedy of the commons
Source: Sci Rep. 2021 Sep 2;11:17585. doi: 10.1038/s41598-021-97001-3 (PMC8413299; doi:10.1038/s41598-021-97001-3)
Supplement: Supplementary file 1 — Supplementary Information. [file 41598_2021_97001_MOESM1_ESM.pdf]

Supplementary Information

A Preliminaries

A.1 Definitions

Let G be a connected, undirected network. In the context of our models, we often refer to the vertices of G as players. Given a player u, we write $N(u)$ the set of *neighbors* of u. Similarly, given a subset A of players, we write $N(A)$ the set of neighbors of A, that is

$$N(A) = \bigcup_{u \in A} N(u).$$

The *degree* of a player u is the number of its neighbors, that is $|N(u)|$. We say that G has minimal degree Δ if every player has degree at least Δ. A network is called Δ-*regular* if all the vertices have degree precisely Δ.

For two players u and v in G, let $d_G(u, v)$ denote the *distance* between u and v, that is, the number of edges on the shortest path linking u to v in G. Similarly, given a subset A of players, we write $d_G(u, A)$ the distance between u and A, that is

$$d_G(u, A) = \min_{v \in A} d_G(u, v).$$

The *diameter* of G, is

$$\mathrm{diam}(G) = \max_{u,v \in G} d_G(u, v).$$

A *bipartite network* is a network G whose set of vertices can be divided into two disjoint sets U and V, such that every edge connects a player in U to a player in V. It is a well-known fact that a network is bipartite network if and only if it does not contain any odd-length cycles [1].

A.2 A result in graph theory

The following lemma (mentioned also in [2]) appears to be a basic result in graph theory, however, we could not find a formal proof for it. We therefore provide a proof here for the sake of completeness.

Lemma 3. *The shortest odd-length cycle of any non-bipartite network G is of length at most $2\mathrm{diam}(G) + 1$.*

Proof. Consider a non-bipartite network G. Such a network necessarily has an odd-length cycle. Let $2k + 1$ be the shortest length among the odd-length cycles in G, where k is an integer, and let $C = (u_1, \ldots, u_{2k+1})$ be such a cycle.

Claim 4. *For every $i, j \in \{1, \ldots, 2k + 1\}$ such that $d_C(u_i, u_j) \geq 2$, there exist $\ell \neq i, j$ and a shortest path P between u_i and u_j such that P contains u_ℓ.*

Proof of Claim 4. Assume by way of contradiction that we can find $i < j$ such that no shortest path between u_i and u_j has any intermediate vertex among $\{u_1, \ldots, u_{2k+1}\}$. Up to re-indexing the vertices of the cycle, we can assume that $j - i \leq k$. Let $(u_i = v_1, v_2, \ldots, v_s, v_{s+1} = u_j)$ be a shortest path between u_i and u_j. By assumption, $\{v_2, \ldots, v_s\} \cap \{u_1, \ldots, u_{2k+1}\} = \emptyset$, and $s < j - i$ (otherwise $(u_i, u_{i+1}, \ldots, u_{j-1}, u_j)$ is a shortest path). Consider two cases:

- If s and $j - i$ have different parities, then $s + j - i$ is odd. Moreover, $s + j - i \leq 2(j - i) \leq 2k$, so

$$(v_1 = u_i, u_{i+1}, \ldots, u_{j-1}, u_j = v_{s+1}, v_s, \ldots, v_2)$$

  is an odd-length cycle shorter than C, which is a contradiction.

- If s and $j - i$ have the same parity, then $2k + 1 + s - (j - i)$ is odd. Moreover, $2k + 1 + s - (j - i) < 2k + 1$, so

$$(u_i = v_1, v_2, \ldots, v_s, v_{s+1} = u_j, u_{j+1}, \ldots, u_{2k+1}, u_1, \ldots, u_{i-1})$$

  is again an odd-length cycle shorter than C, which is a contradiction.

This conclues the proof of Claim 4. ☐

Claim 5. *For every $i, j \in \{1, \ldots, 2k + 1\}$, there exist a shortest path P between u_i and u_j such that P contains only vertices of C – in other words, $d_C(u_i, u_j) = d_G(u_i, u_j)$.*

Proof of Claim 5. We prove the claim by induction on $d_C(u_i, u_j)$, the distance between u_i and u_j in C. When $d_C(u_i, u_j) = 1$, (u_i, u_j) is a path of length 1 between u_i and u_j containing only vertices of C. Next, let us assume that the claim holds for every pair of vertices whose distance in C is at most $1 \leq d \leq k$. Consider i and j such that $d_C(u_i, u_j) = d + 1$. By Claim 4, we can find ℓ and a shortest path P between u_i and u_j such that P contains u_ℓ. By the induction hypothesis, we can find shortest paths P_1 between u_i and u_ℓ, and P_2 between u_ℓ and u_j, such that P_1 and P_2 contain only vertices of C. By merging P_1 and P_2, we obtain a shortest path between u_i and u_j containing only vertices of C, which establishes the induction step. This concludes the proof of Claim 5. ☐

By Claim 5, $k = d_C(u_1, u_{k+1}) = d_G(u_1, u_{k+1}) \leq \text{diam}(G)$, where the last inequality is by the definition of diameter. Hence, $2k + 1 \leq 2\text{diam}(G) + 1$. This concludes the proof of Lemma 3. ☐

B Proof of Theorem 1

The goal of this section is to prove Theorem 1. In fact, we prove the more general theorem below.

Theorem 6. *Consider a network G with n players and minimal degree Δ. Assume that the following conditions hold.*

- Condition (i) $E_h + \rho_h < \rho_d$, *and*
- Condition (ii) $E_h + \rho_h \cdot \Delta > 1$.

Then, for some constant $c > 1$ (that depends only on ϵ and not on G) the following holds.

- *If G is not bipartite then with probability at least $1 - \frac{1}{c^n}$, in at most $3 \cdot \text{diam}(G) + 1$ rounds, the system will be in a configuration in which all players are cooperative, and will remain in this configuration forever.*

- *If G is bipartite and Δ-regular then with probability at least $1 - \frac{1}{c^n}$, in at most* $\mathrm{diam}(G) + 1$ *rounds, the system will be in a configuration in which all players are cooperative, and will remain in this configuration forever.*

- *If G is bipartite then with probability at least $1 - \frac{1}{c^\Delta}$, in at most* $\mathrm{diam}(G) + 1$ *rounds, the system will be in a configuration in which all players are cooperative, and will remain in this configuration forever.*

Before we prove Theorem 6 we note that in the third item, the probability bound of $1 - \frac{1}{c^\Delta}$ is tight for bipartite graphs, up to replacing c with another constant. Indeed, consider the bipartite graph which is constructed by having Δ players in U, each of which is connected to each of the remaining $n - \Delta$ players in V. Then, with probability $\frac{1}{c^\Delta}$, for some constant c, all players in U are defectors initially. In this case, it is possible to show that, regardless of the relationships between ρ_d, ρ_h and E_h, but as long as being a defector is the best choice when all neighbors are defectors, the system keeps alternating forever, so that on even rounds all players in U are defectors, and on odd rounds all players in V are defectors.

Proof of Theorem 6. We start with defining $\bar{D}_t$ as the set of non-defector players at round t. The following lemma describes the propagation of the non-defector state in the network. It says that a player u is a non-defector at round $t+1$ if and only if at least one of its neighbors v is a non-defector in round t. Note, however, that this does not imply that the neighbor v remains a non-defector in the next round as well.

Lemma 7. *Under Condition* (i)*, $\bar{D}_{t+1} = N(\bar{D}_t)$.*

Proof. First, we prove that $N(\bar{D}_t) \subseteq \bar{D}_{t+1}$. Let $u \in N(\bar{D}_t)$. By definition, there exists a neighbor v of u such that v is a non-defector at round t. We claim that for u, being a hypocritical in round $t+1$ is strictly more beneficial than being a defector. Indeed, as a hypocritical it will pay $E_h + \rho_h \cdot \Delta_{\bar{d}}(u)$, and as a defector it will pay $\rho_d \cdot \Delta_{\bar{d}}(u)$. Since v is non-defector then $\Delta_{\bar{d}}(u) \geq 1$, and hence:

$$E_h + \rho_h \cdot \Delta_{\bar{d}}(u) \leq (E_h + \rho_h) \cdot \Delta_{\bar{d}}(u) < \rho_d \cdot \Delta_{\bar{d}}(u),$$

where we used Condition *(i)* in the last inequality. Therefore, the cost of u as a defector is strictly higher than its cost as a hypocritical. This implies that in the next round u will be either a hypocritical or a cooperative player, i.e., $u \in \bar{D}_{t+1}$.

To prove the other inclusion, $\bar{D}_{t+1} \subseteq N(\bar{D}_t)$, consider a player $u \notin N(\bar{D}_t)$, i.e., having only defectors as neighbors at round t, or in other words, at round t, we have $\Delta_{\bar{d}}(u) = 0$. If u chooses to be a defector at round $t+1$, then it would pay $\Delta_{\bar{d}}(u)\rho_d = 0$, which is less than what it would pay as a hypocritical ($E_h + \Delta_{\bar{d}}(u)\rho_h = E_h$) or cooperator (1). Hence, $u \notin \bar{D}_{t+1}$. ◻

Lemma 8. *Assume that Conditions* (i) *and* (ii) *hold, and assume that for some round t_0 all players are non-defectors. Then, at round $t_0 + 1$, all players will be cooperative, and will remain cooperative forever.*

Proof. Assume that at round t_0 all players are non-defectors. By Lemma 7, we know that every player will remain non-defector for every round after t_0. It therefore remains to show, that at the end of round t, for any $t \geq t_0$, being a cooperative is strictly more beneficial than being a hypocritical.

Observe that since each player has at least Δ neighbors, and since all neighbors are non-defectors at round t, then for every player u, we have $\Delta_{\bar{d}}(u) \geq \Delta$ at round t. Therefore, being a hypocritical costs $E_h + \rho_h \cdot \Delta_{\bar{d}}(u) \geq E_h + \rho_h \cdot \Delta$. By Condition *(ii)*, this quantity is strictly greater than 1, hence more than what a cooperative player would pay. It follows that, at the end of round t, being a cooperative is strictly more beneficial than being a hypocritical, implying that all players would be cooperators at round $t+1$. This completes the proof of Lemma 8. ◻

Lemma 9. *Assume that Conditions* (i) *and* (ii) *hold, and assume that for some round t_0, we have $\bar{D}_{t_0} \cap N(\bar{D}_{t_0}) \neq \emptyset$, that is, there are at least two neighboring non-defectors. Then in at most* $\mathrm{diam}(G)+1$ *rounds as of round t_0, the system will be in the configuration in which all players are cooperative, and will remain in this configuration forever.*

Proof. By assumption, there exists two neighbors $u_0, u_0' \in \bar{D}_{t_0}$. We define inductively a sequence of sets $\{U_j\}_{j\geq 0}$, setting $U_0 = \{u_0, u_0'\}$, and for every j, defining $U_{j+1} = N(U_j)$.

Claim 10. *For every integer $j \geq 0$,*

$$U_j \subseteq N(U_j) \tag{3}$$

(each player in U_j has at least one neighbor in U_j), and

$$U_j \subseteq \bar{D}_{t_0+j} \tag{4}$$

(each player in U_j is non-defector at round t_0+j).

Proof of Claim 10. The proof proceeds by induction. The base of the induction, corresponding to $j=0$, is true by the assumption on u_0 and u_0'. Next, let us assume that the claim holds for some integer $j \geq 0$. By the induction hypothesis with respect to Eq. (3), $U_j \subseteq N(U_j)$, so $N(U_j) \subseteq N(N(U_j))$, and hence, by definition, $U_{j+1} \subseteq N(U_{j+1})$. In other words, we have proved that Eq. (3) holds at round $j+1$. Next, by the induction hypothesis with respect to Eq. (4), we have $U_j \subseteq \bar{D}_{t_0+j}$, so $N(U_j) \subseteq N(\bar{D}_{t_0+j})$. By definition of U_{j+1}, and by Lemma 7, we can rewrite this as $U_{j+1} \subseteq \bar{D}_{t_0+j+1}$, establishing Eq. (4) at round $j+1$. This completes the induction step and concludes the proof of Claim 10. □

A direct consequence of Eq. (3) in Claim 10 and the definition of the sequence $\{U_j\}_j$ is that $U_{j+1} = U_j \cup N(U_j)$, and so, U_{j+1} is equal to U_j together with all the neighbors of players in U_j. As a consequence, for every $j \geq \mathrm{diam}(G)$, the set U_j contains all players. By Eq. (4) of Claim 10, this implies that from round $t_0 + \mathrm{diam}(G)$ onward, all players are non-defectors.

By Lemma 8, we conclude that from round $t_0 + \mathrm{diam}(G) + 1$ onward, all players are cooperative. This completes the proof of Lemma 9. □

Lemma 11. *Assume that Conditions* (i) *and* (ii) *hold, and that G is not bipartite. If $\bar{D}_0 \neq \emptyset$, i.e., if initially there is at least one non-defector player, then in at most* $3 \cdot \mathrm{diam}(G) + 1$ *rounds, the system will be in the configuration in which all players are cooperative, and will remain in this configuration forever.*

Proof. By assumption, G is not bipartite, or equivalently, G contains at least one odd-length cycle. Let $(u_1, \ldots, u_{2k+1})$ be a shortest odd-length cycle of G. Given $s = d_G(u_1, \bar{D}_0)$, let $(v_0 \in \bar{D}_0, v_1, \ldots, v_{s-1}, v_s = u_1)$ be a shortest path from $\bar{D}_0$ to u_1. By Lemma 7, it follows by induction that for every $t \in \{0, \ldots, s\}$, $v_t \in \bar{D}_t$, and hence, $u_1 \in \bar{D}_s$ (note that, although $v_{t-1} \in \bar{D}_{t-1}$, it could be that $v_{t-1} \notin \bar{D}_t$). Similarly, for every $t \in \{1, \ldots, k\}$, $u_{1+t} \in \bar{D}_{s+t}$ and $u_{2k+2-t} \in \bar{D}_{s+t}$. Hence, $u_{k+1} \in \bar{D}_{s+k}$ and $u_{k+2} \in \bar{D}_{s+k}$. In other words, we have just showed that in round $s+k$, we have two non-defector neighbors.

By the definition of diameter, $s \leq \mathrm{diam}(G)$. By Lemma 3, we also have $k \leq \mathrm{diam}(G)$. By Lemma 9, the system needs at most $\mathrm{diam}(G)+1$ rounds after round $s+k$ to reach full cooperation. We conclude that it reaches cooperation in at most $3 \cdot \mathrm{diam}(G) + 1$ rounds, as stated. □

Lemma 12. *Assume that Conditions* (i) *and* (ii) *hold, and that G is bipartite. The set of players can be partitioned into U and V such that $U \cap N(U) = V \cap N(V) = \emptyset$. If $\bar{D}_0 \cap U \neq \emptyset$ and $\bar{D}_0 \cap V \neq \emptyset$, then in at most $T = \mathrm{diam}(G) + 1$ rounds, the system will be in the configuration in which all players are cooperative, and will remain in this configuration forever.*

Proof. By assumption, G is bipartite. We define inductively a sequence of subsets of the set of players, $U_0 = U \cap \bar{D}_0$, and for every $k \geq 0$, $U_{k+1} = N(N(U_t))$ – that is, U_{k+1} contains the neighbors (in U) of the neighbors (in V) of the players in U_k. Note that, as a consequence of this definition, $U_k \subseteq U_{k+1}$. Let $k_0 = \lfloor \text{diam}(G)/2 \rfloor$.

Let us show that $U_{k_0} = U$ (and hence, that for every $k \geq k_0$, $U_k = U$). For this purpose, consider a player $u \in U$. Let $(u_0, v_0, u_1, v_1, \ldots, u_{s-1}, v_{s-1}, u_s)$, where $u_0 \in U_0$ and $u_s = u$ be a shortest path from U_0 to u. This path is of length $2s \leq \text{diam}(G)$, so $s \leq k_0$. Since $u_{\ell+1} \in N(N(u_\ell))$ for every $\ell \leq s$, it follows by induction on ℓ that for every $\ell \leq s$, $u_\ell \in U_\ell$, and hence that $u \in U_s$. As we have seen, the sequence $\{U_k\}_k$ is non-decreasing and since $s \leq k_0$, we obtain $u \in U_{k_0}$. This establishes that $U_{k_0} = U$.

Next, we prove by induction that for every k, $U_k \subseteq \bar{D}_{2k}$. This is true for $k = 0$ by definition. Assume that this is true for some integer $k \geq 0$. We have

$$U_{k+1} = N(N(U_k)) \subseteq N(N(\bar{D}_{2k})) = \bar{D}_{2k+2},$$

where the second transition is by the induction hypothesis and the last transition is due to Lemma 7. This concludes the induction proof. Since we have already proved that $U_{k_0} = U$, we conclude that $U \subseteq \bar{D}_{2k_0}$.

We can apply the same reasoning to V and obtain that $V \subseteq \bar{D}_{2k_0}$. Thus, $\bar{D}_{2k_0}$ contains all players. By Lemma 8, from round $2k_0+1 \leq 2\text{diam}(G)+1$ onward, the system will be in a configuration in which all players are cooperative and will remain in this configuration forever. This concludes the proof of Lemma 12. □

Finally, we wrap the aforementioned lemmas to prove the theorem with respect to different networks. Recall that initially, each player is set to be a defector with probability $1 - \epsilon$, a hypocritical with probability $\epsilon/2$, and a cooperative with probability $\epsilon/2$, for some fixed $0 < \epsilon < 1$ independent of n. We consider three families of networks.

- If G is not bipartite, then Lemma 11 guarantees that the system converges to full cooperation in $3 \cdot \text{diam}(G) + 1$ rounds, provided that the initial configuration contains at least one non-defector. This happens with overwhelmingly high probability, specifically, $1 - (1 - \epsilon)^n = 1 - \frac{1}{c^n}$, for some constant $c > 1$. This completes the proof of the first item in Theorem 6.

- If G is bipartite, then the set of players in G can be split into two disjoint sets U and V such that all edges are between U and V. Lemma 12 guarantees that the system converges to full cooperation $\text{diam}(G) + 1$ rounds, provided that there is at least one non-defector in U and at least one non-defector in V. Let us see what is the probability that the initial configuration satisfies this.

  – If G is Δ-regular, then both U and V contain precisely $n/2$ players. This follows from the fact that the number of edges outgoing from U, respectively V, is precisely $\Delta|U|$, respectively $\Delta|V|$, and these numbers are equal. In this case the probability that there is at least one non-defector in U and at least one non-defector in V is $\left(1 - (1 - \epsilon)^{n/2}\right)^2 \geq 1 - 2(1 - \epsilon)^{n/2} > 1 - \frac{1}{c^n}$, for some constant $c > 1$. This completes the proof of the second item in Theorem 6.

  – For general bipartite G with minimal degree Δ, we have that both $|U|$ and $|V|$ are greater or equal to Δ. Hence, the probability that there is at least one non-defector in U and at least one non-defector in V is at least $\left(1 - (1 - \epsilon)^{\Delta}\right)^2 \geq 1 - 2(1 - \epsilon)^{\Delta} > 1 - \frac{1}{c^{\Delta}}$, for some constant $c > 1$. This completes the proof of the third item in Theorem 6.

□

C Proof of Theorem 2

The goal of this section is to prove Theorem 2. In fact, we prove the more general theorem below.

Theorem 13. *Consider a network G with n players and minimal degree Δ undergoing the two-order model, so that the following conditions hold.*

- Condition (i) $\alpha_2 < \beta_2$, *and*
- Condition (ii) $\alpha_1 < \Delta\beta_1$.

Then the following holds for some constant $c > 1$.

- *If G is not bipartite then with probability at least $1 - \frac{1}{c^n}$, in at most $3 \cdot \mathrm{diam}(G) + 1$ rounds, the system will be in a configuration in which all players are cooperative, and will remain in this configuration forever.*
- *If G is bipartite and Δ-regular then with probability at least $1 - \frac{1}{c^n}$, in at most $\mathrm{diam}(G) + 1$ rounds, the system will be in a configuration in which all players are cooperative, and will remain in this configuration forever.*
- *If G is bipartite then with probability at least $1 - \frac{1}{c^\Delta}$, in at most $\mathrm{diam}(G) + 1$ rounds, the system will be in a configuration in which all players are cooperative, and will remain in this configuration forever.*

Before we prove the theorem, we note that Condition (ii) is necessary for the emergence of cooperation on Δ-regular graphs, since having $\alpha_1 > \Delta\beta_1$ would imply that it is always beneficial to defect on the first level.

Proof of Theorem 13. Consider a network G, and parameters α_1, α_2, β_1, and β_2, satisfying Conditions (i) and (ii) in Theorem 13. We first observe that under Condition *(i)*, at any round $t \geq 1$, no player ever chooses to be a private cooperator in G. Indeed, if $\Delta_2(u) \geq 1$, then a private cooperator would pay $\alpha_1 + \Delta_2(u)\beta_2 > \alpha_1 + \Delta_2(u)\alpha_2 \geq \alpha_1 + \alpha_2$, hence, more than the cost of cooperating, and when $\Delta_2(u) = 0$, a private cooperator would pay $\alpha_1 > 0$, hence, more than the cost of defecting. It follows that, although the initial configuration may include private cooperators, this behavior completely disappears from the system after the first round.

Next, we aim to prove Theorem 13 by reducing it to Theorem 6. Let G' be a network identical to G, undergoing the main model (for which Theorem 6 applies), taking the parameters:

$$E_h = \frac{\alpha_2}{\alpha_1 + \alpha_2}, \quad \rho_h = \frac{\beta_1}{\alpha_1 + \alpha_2}, \quad \text{and} \quad \rho_d = \frac{\beta_1 + \beta_2}{\alpha_1 + \alpha_2}. \tag{5}$$

A *configuration* $\mathcal{C}$ on G is an assignment of behaviors, namely, either defectors, cooperators, hypocritical, or private cooperators, to the players in G. Recall that the initial configuration on G is sampled according to the distribution $\psi(\epsilon)$, so that each player is initially chosen to be a defector with probability $1 - \epsilon$, and any of the three remaining behaviors with probability $\epsilon/3$.

We next define a mapping f, transforming each initial configuration $\mathcal{C}$ on G to an initial configuration $\mathcal{C}'$ on G'. The mapping is very simple: All players in G' remain with the same behavior as in G except that private cooperators are turned into defectors. It is easy to see that given the distribution $\psi(\epsilon)$, this mapping induces the distribution $\psi'(\epsilon')$ on the initial configurations in G', where $\epsilon' = \frac{2}{3}\epsilon$. Indeed, under this mapping, a player in G' is initially chosen to be a defector with probability $1 - \epsilon + \epsilon/3 = 1 - \epsilon'$, a cooperator with probability $\epsilon/3 = \epsilon'/2$, and hypocritical with probability $\epsilon/3 = \epsilon'/2$.

At this point, we address a technicality that concerns the randomness involved in breaking ties. That is, recall that at any round t, if the minimal cost is attained by several behaviors then the player chooses one of them uniformly at random. One way to implement this is by considering a certain order between the behaviors, and sampling a number uniformly at random $r \in [0, 1]$. For instance, consider the following ordering: cooperator > hypocritical > defector (as we saw, in the regime of parameters we consider, a private cooperator in the two-order model never attains the minimal cost, and hence it is never considered as an option). If a player needs to choose, say, between being a cooperator or a defector, then it chooses to be a cooperator if r is in $[0, 0.5]$, and otherwise, it chooses to be a defector. This means that given a sequence of random numbers $\{r_i\}_{i=1}^{\infty}$, where $r_i \in [0, 1]$, the behavior of a player is deterministically described by the behaviors of its neighbors at each round.

Consider a fixed sequence of random numbers $\{r_i\}_{i=1}^{\infty}$, where $r_i \in [0, 1]$. Let $\mathcal{C}_0$ be an initial configuration in G, and let $\mathcal{C}_t$ denote the configuration $\mathcal{C}_0$ at round t, with the costs defined according to the two-order model on G, and using the sequence $\{r_i\}_{i=1}^{\infty}$ to break ties if necessary. Let $\mathcal{C}'_0 = f(\mathcal{C}_0)$ be the mapped configuration on G', and let $\mathcal{C}'_t$ be the corresponding configuration at round $t \geq 1$, with the costs defined according to the parameters mentioned in Eq. (5), and using the same sequence $\{r_i\}_{i=1}^{\infty}$ to break ties if necessary.

Claim 14. *For every $t \geq 1$, we have*

$$\mathcal{C}'_t = \mathcal{C}_t,$$

Proof of Claim 14. Our goal is to show that at any round $t \geq 0$, a player u in $\mathcal{C}_t$ is a defector, a cooperator or a hypocritical, respectively, if and only if it is a defector, a cooperator or a hypocritical, respectively, in $\mathcal{C}'_t$, and that a private cooperator in $\mathcal{C}_t$ is a defector in $\mathcal{C}'_t$.

Let us prove this claim by induction. By definition, the claim holds for $t = 0$. Assume that it holds for some integer $t \geq 0$. By the induction hypothesis, for every player u, the set $\Delta_2(u)$ in G is equal to $\Delta_{\bar{d}}(u)$ in G'. Hence, with our definitions of E_h, ρ_h and ρ_d in Eq. (5), we argue that the cost of being a cooperator in $\mathcal{C}'_t$ (respectively hypocritical, defector) is $\frac{1}{\alpha_1+\alpha_2}$ times the cost of being a cooperator in $\mathcal{C}_t$ (respectively hypocritical, defector). Indeed, a cooperator in $\mathcal{C}'_t$ pays

$$1 = \frac{1}{\alpha_1 + \alpha_2} \cdot (\alpha_1 + \alpha_2),$$

while $(\alpha_1 + \alpha_2)$ is what it pays in $\mathcal{C}_t$. A hypocritical player in $\mathcal{C}'_t$ pays

$$E_h + \Delta_{\bar{d}}(u) \cdot \rho_h = \frac{\alpha_2}{\alpha_1 + \alpha_2} + \Delta_2(u) \frac{\beta_1}{\alpha_1 + \alpha_2},$$

while $(\alpha_2 + \Delta_2(u) \cdot \beta_1)$ is what a hypocritical pays in $\mathcal{C}_t$, and a defector in $\mathcal{C}'_t$ pays

$$\Delta_{\bar{d}}(u) \cdot \rho_d = \Delta_2(u) \cdot \frac{\beta_1 + \beta_2}{\alpha_1 + \alpha_2},$$

while $\Delta_2(u) \cdot (\beta_1 + \beta_2)$ is what it pays in $\mathcal{C}_t$.

Moreover, recall that no player in G ever chooses to be a private cooperator in rounds $t \geq 1$. Hence, the behavior that minimizes the cost in G is the same as in G'. It follows that at round $t + 1$, all players choose the same behavior in G as they would in G', which establishes the induction proof, and concludes the proof of Claim 14. □

Next, we prove that with our choices of E_h, ρ_h and ρ_d in Eq. (5), Conditions *(i)* and *(ii)* in Theorem 13

imply Conditions *(i)* and *(ii)* in Theorem 6:

$$\begin{aligned}\alpha_2 < \beta_2 &\iff \alpha_2 + \beta_1 < \beta_1 + \beta_2 \\ &\iff \frac{\alpha_2}{\alpha_1+\alpha_2} + \frac{\beta_1}{\alpha_1+\alpha_2} < \frac{\beta_1+\beta_2}{\alpha_1+\alpha_2} \\ &\iff E_h + \rho_h < \rho_d,\end{aligned}$$

and

$$\begin{aligned}\Delta \cdot \beta_1 > \alpha_1 &\iff \alpha_2 + \Delta \cdot \beta_1 > \alpha_1 + \alpha_2 \\ &\iff \frac{\alpha_2}{\alpha_1+\alpha_2} + \Delta \cdot \frac{\beta_1}{\alpha_1+\alpha_2} > 1 \\ &\iff E_h + \rho_h \cdot \Delta > 1.\end{aligned}$$

Hence, we can apply Theorem 6 to the mapped process on G'. It follows that in the number of rounds and probability guarantees as stated in Theorem 6, G' converges to the configuration in which all players are cooperators, and remains in that configuration forever. By Claim 14, this holds for G as well, concluding the proof of Theorem 13. □

References

[1] Reinhard Diestel. Graph theory 3rd ed. *Graduate texts in mathematics*, 173, 2005.

[2] Ali Mohammadian. A generalization of the odd-girth theorem. *Discrete Mathematics*, 339(2):1052–1057, 2016.
